# Occupational diseases in Murmansk Oblast: 1980–2010

**DOI:** 10.3402/ijch.v72i0.20468

**Published:** 2013-03-19

**Authors:** Alexey A. Dudarev, Liudmila V. Talykova, Jon Øyvind Odland

**Affiliations:** 1 Hygiene Department, Northwest Public Health Research Centre, St. Petersburg, Russia; 2 Kola Research Laboratory for Occupational Health, Kirovsk, Murmansk Oblast, Russia; 3 AMAP Secretariat, University of Tromsø, Tromsø, Norway

**Keywords:** occupational diseases, labour conditions, attestation of workplaces, medical examination, musculoskeletal, respiratory, nervous system, hearing loss, vibration disease, Russian Arctic, Murmansk Oblast

## Abstract

**Background:**

Official statistics tend to underestimate the incidence of occupational disease (OD) nationally and regionally in Russia.

**Objectives:**

The general aim was to obtain an accurate estimate of ODs in Murmansk Oblast in 1980–2010 and to determine the rate of specific types of ODs among cohorts of workers who had been exposed to the hazardous factors causing the disease.

**Materials and methods:**

Data were retrieved from the Murmansk Oblast ODs database for the oblast and 2 enterprises – Apatite JSC and Kolskaya MSC – which contributed to more than half of the ODs in the oblast in 1980–2010. The total number of ODs and 5 specific categories (musculoskeletal, respiratory, nervous diseases, hearing loss and vibration disease) were analysed.

**Results:**

The total rate of ODs among workers of main shops in both enterprises who were actually exposed to harmful factors were extremely high: the rate for Apatite JSC was 25 times higher than in Russia and 15 times higher than in Murmansk Oblast, while the rate for Kolskaya MSC was about 30 and 20 times greater than in Russia and in Murmansk Oblast, respectively; in the 2000s the difference reached 100–150 times. The rise in reported ODs in both enterprises corresponded to the time when intensive medical examinations were conducted by the Kola Research Laboratory for Occupational Health (KRLOH) in Kirovsk. A similar pattern was also observed for the sub-categories of musculoskeletal, respiratory, nervous diseases, hearing loss and vibration disease. It is likely that the true burden of OD is even higher due to misdiagnosis, reluctance of workers concerned about job security to present for care and the lack of reliable information on working conditions needed to establish a causal link between disease and occupational exposure.

**Conclusions:**

As with many other regions across Russia, ODs in Murmansk Oblast are grossly underestimated. Serious problems exist in the Russian occupational health care system and the collection of occupational health statistics that require urgent, fundamental reform.

As discussed in the first article in this Special Issue, official statistics in Russia and its regions grossly underestimate the level of occupational diseases (ODs).

The aim of this study was to obtain the “true picture” of ODs in Murmansk Oblast in 1980–2010, using the Murmansk Oblast ODs data base, by matching the number of specific OD types with the number of workers exposed to specific hazardous factors causing the disease, to assess the actual level of specific ODs among specifically exposed cohorts and to compare the results with official statistics data.

## Materials and methods

Murmansk Oblast ODs data base was created in the Centre of Occupational Pathology of Murmansk Oblast based on data collected by the Kola Research Laboratory for Occupational Health (KRLOH) in Kirovsk city of Murmansk Oblast. Since 1962, KRLOH has been responsible for establishing the occupational cause of illness and assigning OD diagnoses in Murmansk Oblast; all enterprises of Murmansk Oblast are obliged to send their workers to KRLOH for assessment.

When an OD is suspected in a polyclinic in the place of residence of a worker, or in the course of periodic medical examination of workers conducted at the industrial enterprises, the patient is referred to KRLOH for assessment, which includes reviewing the hygienic characteristic of the workplace (prepared by the local unit of the state sanitary service), detailed medical examination by specialists in occupational medicine, and a battery of clinical and laboratory tests.

The computer database was generated in 2001. It contains all confirmed ODs in Murmansk Oblast retrospectively to 1943, and is updated annually. For the period 1980–2010, the database contains the records of 3,575 persons, with an average age of 48.4 years at registration, and an average duration of work of 15–20 years. Information on each patient includes name, age, gender, enterprise, shop (workplace), occupation, occupational experience, date of OD registration, diagnoses (may be several), terms of examinations and treatment, and nature of disability.

In this assessment of 30 years of ODs in Murmansk Oblast, OD cases were grouped into 5 main categories (Table I) and averaged over 5-year periods.

**Table I:** Occupational diseases in Murmansk Oblast database, grouped into 5 categories

Group	Occupational diseases	ICD-10 code
1	Vibration disease	T75.2
2	Noise related	
	Sensorineural hearing loss	H90
	Noise effects on inner ear	H83.3
3	Nervous diseases	
	Nerve, nerve root and plexus disorders	G50-G59
	Peripheral neuropathies	G60-G64
	Diseases of myoneural junction and muscle	G70-G73
	Dorsalgia	M54
4	Respiratory diseases	
	Chronic rhinitis, nasopharyngitis and pharyngitis	J31
	Chronic laryngitis and laryngotracheitis	J37
	Unspecified chronic bronchitis	J42
	Bronchial asthma	J45
	Lung diseases due to external agents	J60-J70
	Other interstitial pulmonary diseases	J84
5	Musculoskeletal diseases	
	Arthrosis	M15-M19
	Other joint disorders	M20-M25
	Spinal osteochondrosis	M42
	Spondylopathies	M45-M49
	Other dorsopathies	M50-M54
	Disorders of muscles	M60-M63
	Disorders of synovium and tendon	M65-M68
	Other soft tissue disorders	M70-M79
	Osteonecrosis	M87

**Fig. 1:**
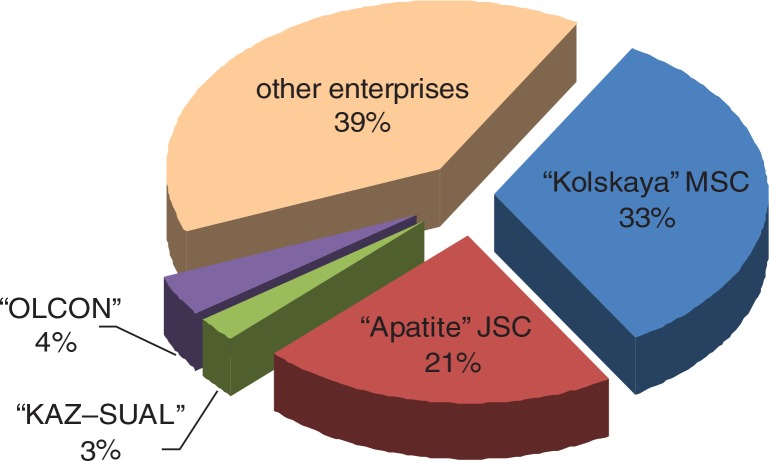
Distribution of patients with diagnosis “OD” among enterprises of Murmansk Oblast in 1980–2010.

Preliminary analysis of the database revealed that 2 enterprises contributed more than half of all ODs cases in Murmansk Oblast during the 30-year period. They are Apatite JSC and Kolskaya MSC (Fig. 1).

Rather than reviewing the entire database, we decided to focus on data from these 2 enterprises only. The following additional types of data were also obtained for analysis:

- number of workers in mines and shops (separate from office workers);
- number of workers exposed to different hazardous factors (hygienic evaluation of workplaces);
- medical examination history of workers;
- information on introduction of new equipment and new technologies;
- information about change of management, replacement of medical experts and any unusual political or economic changes.

Kolskaya MSC is an enterprise for extraction and processing of sulphide copper-nickel ores in the Kola Peninsula, around the settlements of Nickel and Zapolarniy, and in Norilsk. Kolskaya MSC includes the Severonickel industrial complex (which averaged some 13,000 workers over the 30 years) and the Pechenganickel industrial complex (with an average workforce of about 7,000). These industrial complexes carry out extraction and ore processing. Further stages of refinement of nickel and copper are carried out in Monchegorsk city.

Apatite JSC, with about 20,000 workers, is a mining-chemical complex composed of several mines for extraction and 3 concentrating factories for processing of apatite-nepheline raw materials close to Kirovsk city.

The KRLOH has carried out the evaluation of hygienic working conditions occasionally in some shops of Apatite JSC since the 1960s, and in Kolskaya MSC since the mid-1970s. The KRLOH has also carried out investigations at both enterprises since the mid-1990s, as part of the obligatory evaluation process, whereby workplaces are graded as to whether they are optimal, acceptable or hazardous.

The work environment in the main shops of Apatite JSC and Kolskaya MSC could be characterised as substandard and hazardous. Occupational hygiene limits (e.g. on vibration, noise, air dust and chemical agents, and microclimate) are routinely exceeded manifold. Little improvements have occurred over the 30-year study period (1980–2010). The only exception is the vibration factor (and partially hard manual job) in underground mines which have significantly lowered after 2002 as a result of purchasing modern foreign machines.

The KROLH has carried out medical examination of workers in Apatite JSC since the mid-1990s, and in Kolskaya MSC since 2004. Prior to that, episodic medical examinations of workers were conducted as part of some scientific research projects.

The next step of our study was to match the number of specific OD type with the number of workers exposed to the specific hazardous factor causing the disease and to assess the actual level of specific ODs among specifically exposed cohorts.

## Results and discussion

**Fig. 2:**
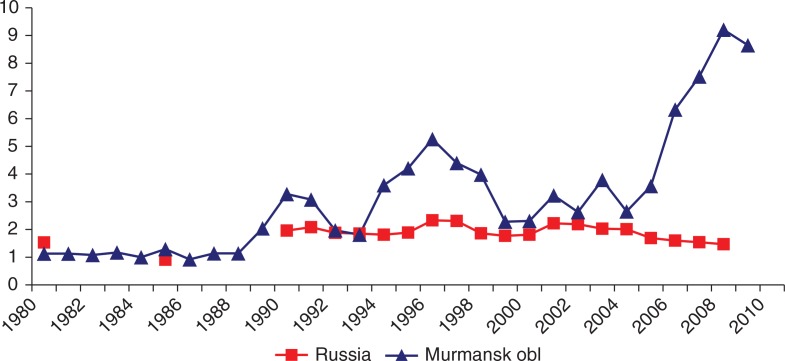
Rate of new cases of occupational diseases (including poisonings) per 10,000 workers in Murmansk Oblast compared to Russia, 1980–2009.

The rate of ODs in Murmansk Oblast based on the KRLOH database over the 30-year study period is compared with the Russian national official statistics in Fig. 2.

The observed increase in Murmansk Oblast is likely the result of more intensive monitoring of workers with detailed medical examinations. This phenomenon is observed elsewhere in Russia, and is discussed in the first article of this Special Issue.

**Fig. 3:**
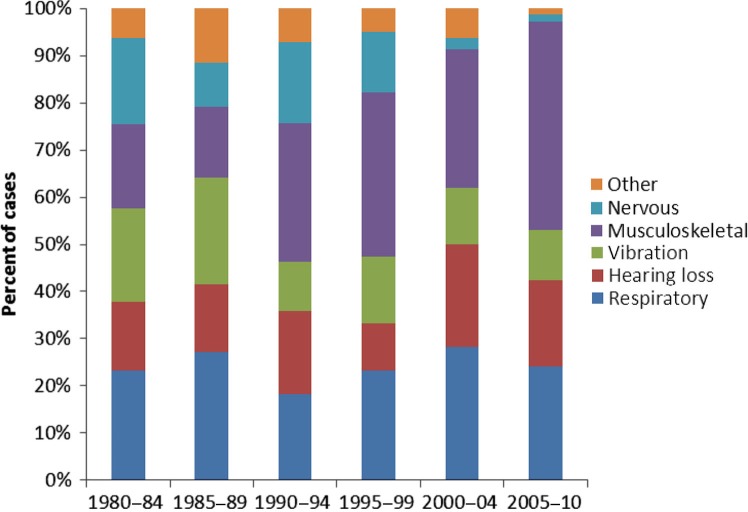
Change in relative proportions of occupational diseases by diagnostic categories.

The changes in the relative proportion of ODs according to diagnostic groups are shown in Fig. 3. The proportion of musculoskeletal diseases among all ODs increased significantly from 15 to 18% in the 1980s, to 35% in 1990s and 44% in 2000s. There was a corresponding decrease in the share of nervous system diseases. Vibration disease also decreased from about 20% in the 1980s to 10–12% in the 2000s. Respiratory diseases and hearing loss remains relatively stable.

**Fig. 4:**
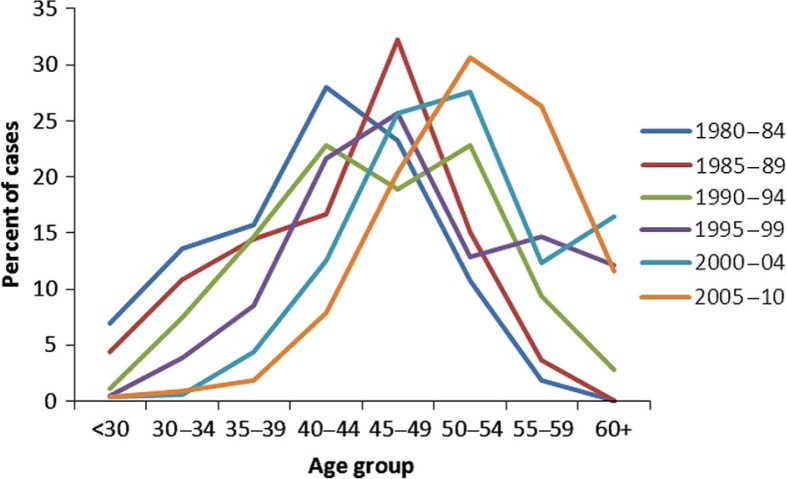
Age distribution of patients with occupational diseases in Murmansk Oblast, 1980–2010.

The age distribution of patients with OD diagnosis is shown in Fig. 4. There appears to be a shift towards a higher proportion of older patients. Possible explanations include the ageing of the work force and reluctance of younger workers with signs and symptoms of OD to present for care.

**Fig. 5:**
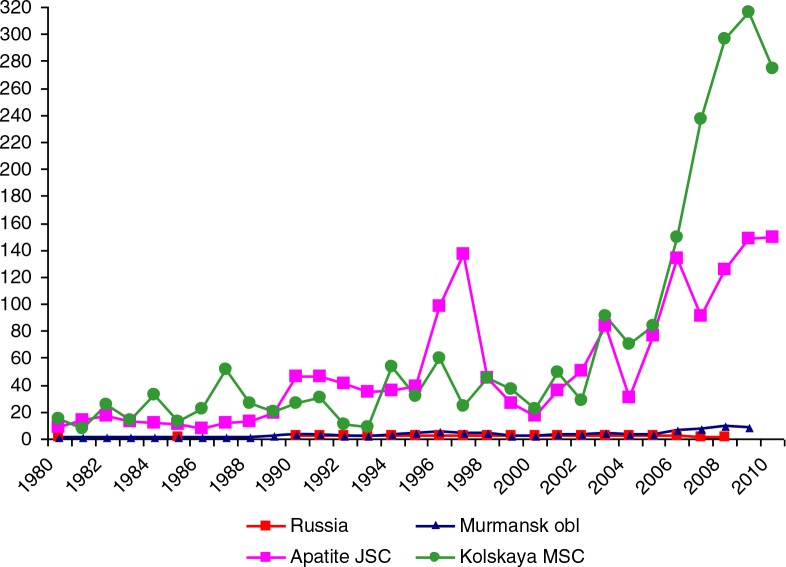
Rates of occupational diseases (per 10,000 workers) in Apatite JSC, Kolskaya MSC, Murmansk Oblast and the Russian Federation, 1980–2010.

In presenting the rate of ODs in Apatite JSC and Kolskaya MSC, we used the actual population at risk, that is, the total number of workers exposed to one or more specific hazardous factors, as the denominator. This amounts to 6,000–9,000 workers in Apatite JSC and 7,000–10,000 workers in Kolskaya MSC during the study period. In Fig. 5 the rate of new ODs (including poisonings) in Apatite JSC and Kolskaya MSC (per 10,000 main shops workers) is compared to Murmansk Oblast based on the Kirovsk database, and official statistics for Russia nationally.

It is evident that the OD levels in both enterprises are extremely high compared to Murmansk Oblast and Russia. Averaged over the 30 years, the rate in Apatite JSC was 25 times higher than in Russia and 15 times higher than in Murmansk Oblast; for Kolskaya MSC it was 30 and 20 times greater than in Russia and Murmansk Oblast, respectively. Considering only the 2000s, the difference reached 100–150 times. Much of the recent increase in Kolskaya MSC since 2006 can be attributed to the start of medical examination of workers by KRLOH experts who have special expertise in occupational medicine than regular primary care doctors that the workers consult. For Apatite JSC, an increase can be observed already in 1996–1997, about the same time when KRLOH specialists began full-fledged examination of workers. In 1998, administrative and staff changes (including resignation of the chief physician) at KRLOH led to the decline in the quality and quantity of services provided, a situation not rectified until the early part of the 2000s.

It should be noted that the Murmansk Oblast data and Russian national data use all workers as the denominator, the majority of whom may not have any exposure to workplace hazards. The number of workers in Kolskaya MSC and Apatite JSC was no more than 10% of the total number of workers in Murmansk Oblast, but they contributed to more than 50% of the reported ODs.

There are some differences between the pattern of ODs in Apatite JSC and Kolskaya MSC. Musculoskeletal diseases, hearing loss and vibration disease are predominant in Apatite JSC while respiratory diseases constitutes up to 50–55% of cases in Kolskaya MSC.

**Fig. 6:**
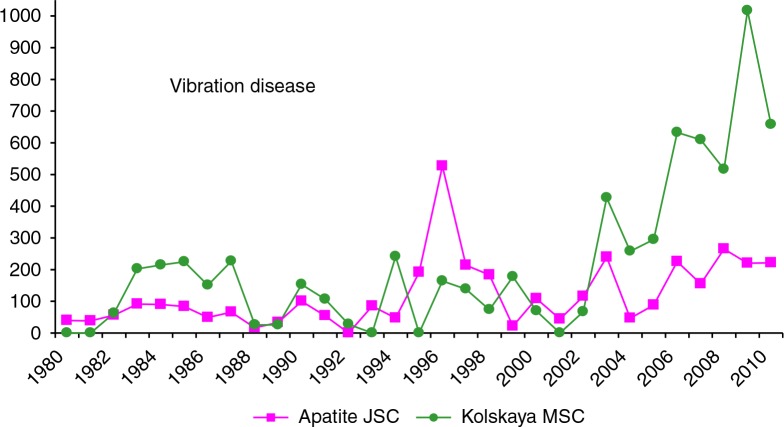
Rate of new cases of vibration disease in Apatite JSC and Kolskaya MSC (per 10,000 workers exposed to local vibration), 1980–2009.

The number of workers exposed to local vibration was 700–1,000 in Apatite JSC and 300–400 in Kolskaya MSC. The rate of vibration disease per 10,000 workers exposed for the 2 enterprises are shown in Fig. 6. Again, the beginning of involvement of KRLOH in intensive examinations corresponded to rises in rates in Kolskaya MSC in the mid-2000s and the mid-1990s in Apatite JSC. Gradual transition to modern foreign machines (with low vibration) began in 2002, but these have not completely replaced manual drilling, which uses old, hard vibrating equipment, and vibration remains a health hazard for these workers.

Hard physical work in conditions of general vibration, low temperature and high humidity results in diseases of the musculoskeletal system, especially those affecting the vertebral column and joints. Quite often doctors in municipal polyclinics attribute musculoskeletal diseases to old age, and not to occupational risk factors, such as a fixed or compelled pose, prolonged lifting and moving of heavy weights. Musculoskeletal pathology is closely connected with disorders of the peripheral nervous system, so diagnosis of musculoskeletal system OD is frequently placed under the category of nervous system ODs, and vice versa.

**Fig. 7:**
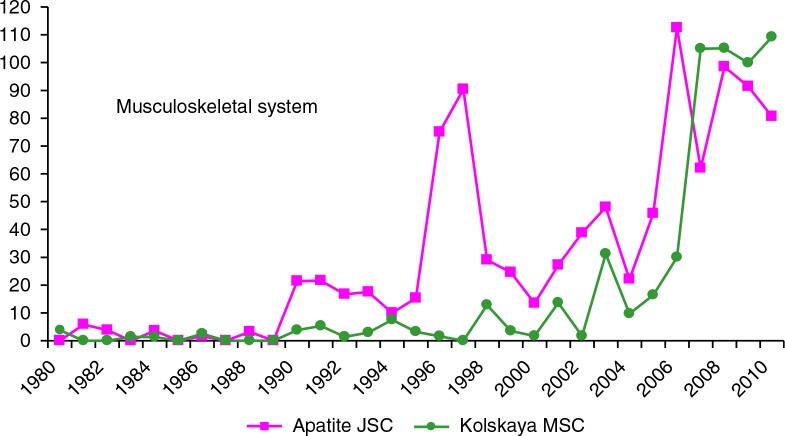
Rate of new cases of musculoskeletal diseases in Apatite JSC and Kolskaya MSC (per 10,000 workers exposed to heavy physical work), 1980–2009.

The number of workers engaged in heavy physical work during 1980–2009 was 4,000–6,000 in Apatite JSC, and 5,500–8,000 in Kolskaya MSC. Both enterprises showed very low levels (or absence) of musculoskeletal diseases in the 1980s. Among Apatite JSC workers, there was a spike in the mid-1990s, and in both enterprises there were increases starting in the mid-2000s (Fig. 7). The trend for nervous system ODs is very similar to musculoskeletal ODs.

**Fig. 8:**
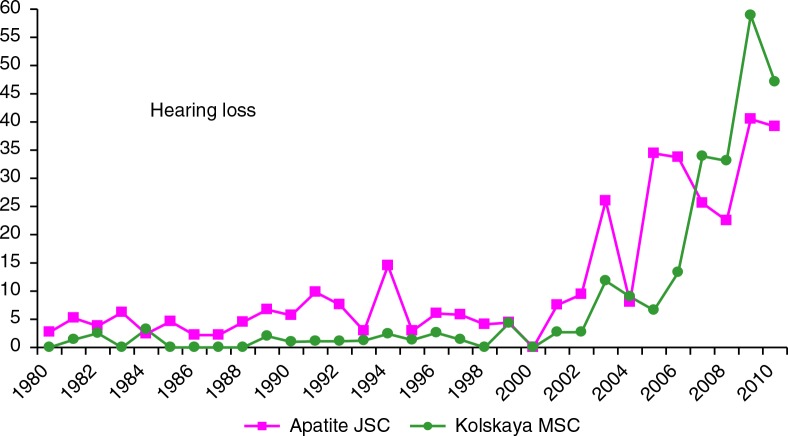
Rate of new cases of hearing loss in Apatite JSC and Kolskaya MSC (per 10,000 workers exposed to noise), 1980–2009.

Regarding noise, it is necessary to note that almost all workers employed at the main shops of both enterprises were (and are) exposed to high levels of this factor. The number of workers exposed to noise during 1980–2009 in Apatite JSC was 6,000–9,000, and it was 7,000–10,000 in Kolskaya MSC. For both enterprises, there was a sharp rise in the 2000s, likely due to improvement in detection (Fig. 8).

**Fig. 9:**
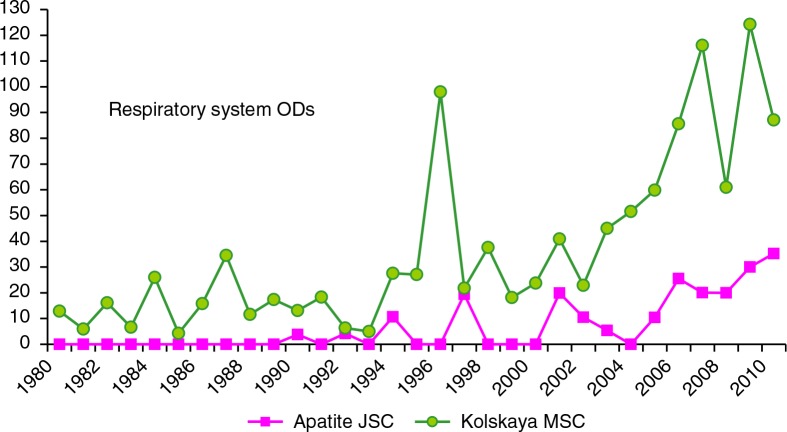
Rate of new cases of respiratory diseases in Apatite JSC and Kolskaya MSC (per 10,000 workers exposed to particulate and chemical air pollution), 1980–2009.

The number of workers exposed to particulate and chemical air pollution during 1980–2009 was 2,000–2,500 workers in Apatite JSC, and 5,000–7,000 in Kolskaya MSC. In Kolskaya MSC, there were high concentrations of nickel and other hazardous chemical air pollutants in the basic shops, and the rate of respiratory ODs in Kolskaya MSC always exceeded that in Apatite JSC. A short-lived peak in 1996 in Kolskaya MSC was due to the appointment of a pulmonologist in the medical-sanitary unit of the enterprise, but the unit was closed and the pulmonologist departed. The increase in both enterprises in the 2000s was due to increased diagnoses by KRLOH (Fig. 9).

Although obligatory certification of workplaces was introduced in Russia in the mid-1990s, the quality and objectivity of the process influences the number of officially approved OD and their occupational origin. Employers are interested in good reports, and, often, a poor report will result in the hiring of another firm of inspectors who are more likely to be sympathetic and not so stringent in the evaluation. Many such firms exist in Karelia, Moscow, St. Petersburg, Arkhangelsk, and so on catering to the lucrative market in Murmansk Oblast.

## Conclusions

The objective of this study was to obtain a truer picture of ODs in Murmansk Oblast than those available from official statistics, making use of the Murmansk Oblast ODs data base. The number of specific OD type was matched with the number of workers exposed to a specific hazardous factor causing the disease to assess the actual level of ODs among specifically exposed cohorts. Detailed analyses were conducted on data from 2 enterprises – Apatite JSC and Kolskaya MSC, which contributed to more than half of ODs of Murmansk Oblast in 1980–2010.

Averaged over a 30-year period, the rate of ODs in Apatite JSC were 25 times higher than in Russia and 15 times higher than in Murmansk Oblast, whereas in Kolskaya MSC the rates were about 30 and 20 times greater than in Russia and Murmansk Oblast, respectively. In both enterprises, there was a notable rise in reported ODs beginning from the years when experienced experts from KRLOH started medical examination of workers. A similar pattern was observed for different categories of ODs – regarding musculoskeletal, respiratory, nervous diseases, hearing loss and vibration disease. The burden of ODs is likely even higher as some cases may have been misdiagnosed, or deliberately hidden by the workers who might be concerned about their job security. There is also a lack of reliable information on working conditions needed to firmly establish linkage between disease and occupational exposure, even when the clinical features are obvious.

This study of one Arctic region in Russia supports the conclusions of the broader survey of Russia's occupational health care system and the underdeveloped state of occupational health statistics described in the overview article. Reform is clearly needed.

